# Influence of the starting day of luteal phase stimulation on double stimulation cycles

**DOI:** 10.3389/fendo.2023.1216671

**Published:** 2023-07-13

**Authors:** Ana Fuentes, Cristina García-Ajofrín, Ruth Romero, Juan Carlos Castillo, Jose A. Ortíz, Mónica Hortal, Jaime Guerrero, Andrea Bernabeu, Rafael Bernabeu

**Affiliations:** ^1^ Department of Reproductive Medicine, Instituto Bernabeu, Alicante, Spain; ^2^ Department of Reproductive Medicine, Instituto Bernabeu Madrid, Madrid, Spain; ^3^ Cátedra de Medicina Comunitaria y Salud Reproductiva, Universidad Miguel Hernández, Elche, Spain; ^4^ Department of Molecular Biology, Instituto Bernabeu BIOTECH, Alicante, Spain; ^5^ Department of Embryology, Instituto Bernabeu, Alicante, Spain

**Keywords:** double stimulation, luteal phase stimulation, DuoStim, oocyte yield, start of stimulation

## Abstract

**Background:**

Double ovarian stimulation is one of the most used strategies in poor-prognosis patients. There is a high heterogeneity between the studies regarding the execution of this stimulation protocol. The aim of this study was to investigate whether the day on which luteal phase stimulation begins after the first oocyte retrieval affects ovarian response in DuoStim cycles.

**Methods:**

This observational and retrospective study included 541 DuoStim cycles between January 2018 and December 2021 in a private fertility clinic. Patients were assigned to 4 groups according to the timing of the onset of luteal phase stimulation after oocyte retrieval (0-2^nd^ day, 3^rd^ day, 4^th^ day and 5^th^-6^th^ day). The primary outcome was the number of oocytes retrieved in the luteal phase in each group.

**Results:**

No differences were found between groups in the number of oocytes collected (5.12 ± 3.56 vs. 5.39 ± 3.74 vs. 5.61 ± 3.94 vs. 5.89 ± 3.92; p=0,6), MII or number of follicles. An increase in the duration of stimulation was found when stimulation started on the 4^th^ day (10.42 ± 2.31 vs. 10.68 ± 2.37 vs. 11.27 ± 2.40 vs. 10.65 ± 2.37 days, p=0,033). A lower number of fertilized oocytes was observed when stimulation began before the fourth day (3.36 ± 2.80 vs. 3.95 ± 2.53 vs. 4.03 ± 2.73 vs. 4.48 ± 3.11; p=0,036). The number of blastocysts was higher when the stimulation started 5-6 days after retrieval (1.82 ± 1.74 vs. 2.13 ± 1.61 vs. 2.33 ± 2.06 vs. 2.91 ± 2.39; p= 0,030).

**Discussion:**

The number of oocytes retrieved does not differ depending on the day that stimulation begins. However, oocytes competence in terms of fertilized oocytes and blastulation, appears to be lower when the second stimulation starts before the fourth day after oocyte retrieval.

## 1 Introduction

The current knowledge about the presence of multiple follicular waves during a single menstrual cycle (1, 2) is the basis for new unconventional IVF protocols aimed at maximizing ovarian response in poor-prognosis patients. One such approach is double ovarian stimulation (DuoStim), which combines follicular phase stimulation (FPS) and luteal phase stimulation (LPS) in the same ovarian cycle.

In 2014, Kuang et al. proposed a double stimulation in the same ovarian cycle with the so-called Shanghai protocol and suggested that a higher number of oocytes could be obtained within a short period of time (3). Since then, several studies have confirmed that double stimulation increases the total number of oocytes compared to the standard single stimulation (4, 5). Ubaldi et al., in 2016, performed a study comparing FPS and LPS in DuoStim protocol and found a similar number of oocytes and blastocysts between stimulations, with an increase in final transferable blastocysts per ovarian cycle (6). Other researches, however, found that cohorts of oocytes obtained in the luteal phase after a first follicular were larger (7–10). These studies also showed that luteal phase oocytes had a similar competence to those from follicular phase, in terms of blastulation and euploidy rate.

Since DuoStim maximizes the number of oocytes collected in a short timeframe, this protocol can be considered in certain groups of patients, including those with low ovarian reserve, advanced maternal age and women who require urgent fertility preservation for medical reasons (6, 11).

The DuoStim protocol consists of a follicular phase stimulation followed by a second stimulation after the first egg retrieval without waiting for a new follicular phase. However, there is a high heterogeneity between the studies regarding the type of gonadotropins used, the dose and the trigger methods. In addition, differences have been observed in the timing of the onset of the second stimulation, which varies between 0 and 7 days after oocyte retrieval (3, 6, 8, 11, 12). The aim of this study is to investigate whether the day of LPS initiation after the first egg retrieval affects the luteal stimulation response in DuoStim cycles.

## 2 Materials and methods

### 2.1 Patients

This is an observational and retrospective study conducted between January 2018 and December 2021. 541 patients treated with a DuoStim protocol in a private fertility clinic were included. The LPS data were traced in the clinical database.

DuoStim was suggested as part of the daily routine in the clinic to patients with low ovarian reserve (according to Bologna criteria), advanced maternal age, and patients without blastocysts in previous cycles, so as to increase the number of oocytes and embryos for transfer or biopsy. Written informed consent was obtained prior to the procedure.

**Figure 1:**
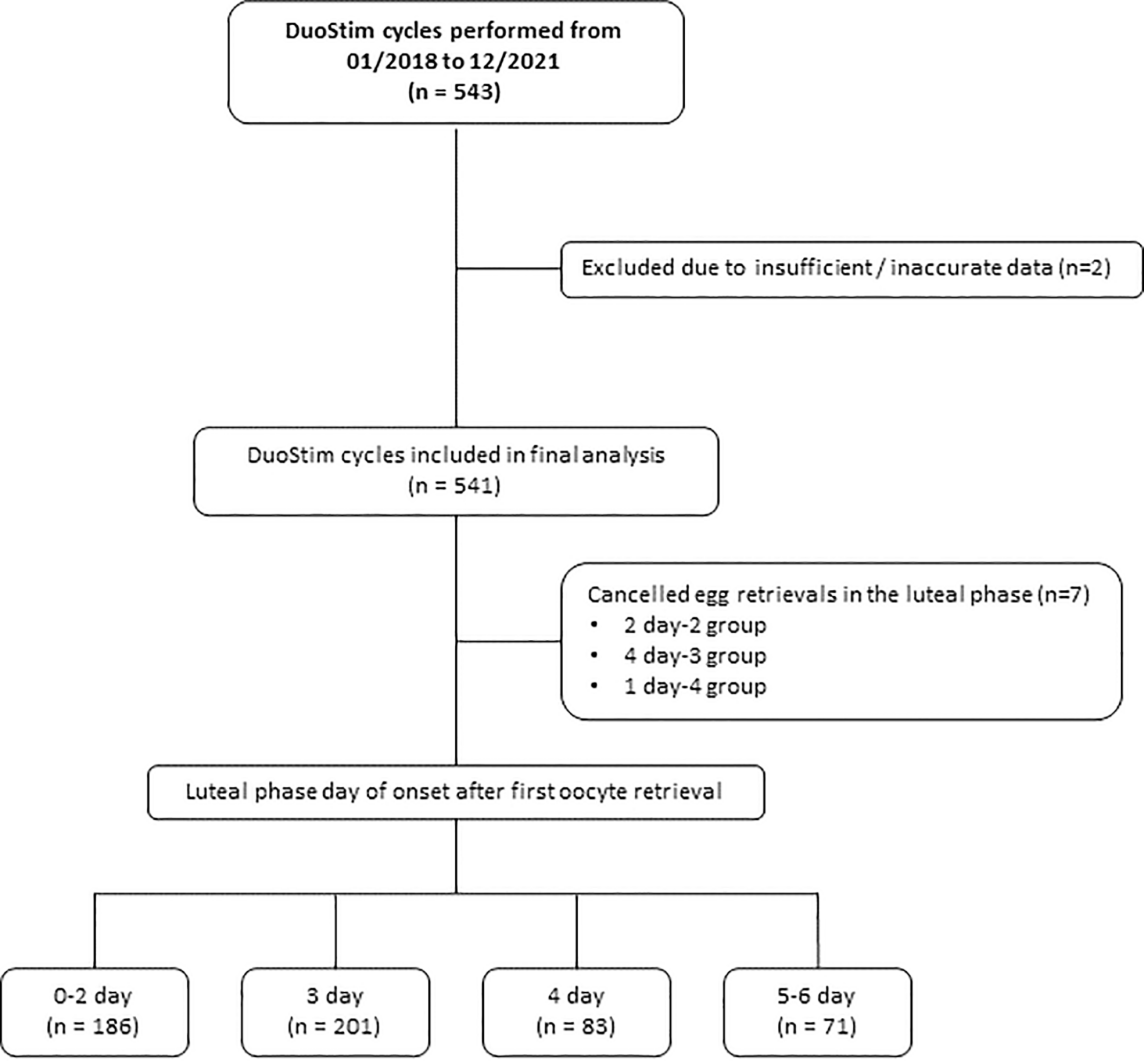
Flowchart.

Data from luteal phase stimulations were compared according to the day of onset after the first oocyte retrieval. For this purpose, the cases were divided into four groups depending on the day on which stimulation began after the first retrieval: 0-2 days group, 3 days group, 4 days group and 5-6 days group. Due to an inadequate response, the scheduled egg retrieval had to be cancelled in a total of 7 patients, with the distribution as follows: two cases on day-2, four cases on day-3, and one case on day-4. However, considering the low number of affected individuals, it was decided to include them in the final analysis. **Figure 1**.

### 2.2 Ethical approval

The design of this study was approved by the Institutional Review Board of the clinic. Code: IBMR42 (June 2022).

### 2.3 Stimulation protocol

Both stimulations were performed with recombinant follicle stimulation hormone (rFSH) or highly purified FSH (HP-FSH) and human menopausal gonadotropin (hMG). The type of gonadotropin and initial dose depended on the age, hormone levels, antral follicular count and the results obtained in previous cycles, if any.

After basal assessment of the ovaries, follicular phase stimulation (FPS) was initiated between the second and fourth day of the menstrual cycle. Daily administration of a gonadotrophin-releasing hormone antagonist (cetrorelix or ganirelix 0.25 mg) was started when the leading follicle had a diameter ≥ 13-14 mm until the day of ovulation trigger. When at least two follicles had reached 17-18 mm in diameter, ovulation was triggered with a subcutaneous bolus of triptorelin at the dose of 0.2 mg (Decapeptyl^®^, Ipsen Pharma, Spain), and oocyte retrieval was performed 36 hours later.

Luteal phase stimulation was initiated between 0 and 6 days after the first oocyte retrieval, but the specific starting day was determined by medical preference and/or organizational reasons, rather than a specific medical criterion. Prevention of LH surge during the luteal phase was performed with 200 mg oral micronized progesterone daily. When at least two follicles had reached 17-18 mm in diameter, ovulation was double triggered with a subcutaneous bolus of triptorelin 0.2 mg (Decapeptyl^®^ Ipsen Pharma, Spain) and a 6500 UI bolus of human chorionic gonadotrophin (Ovitrelle^®^ 250, Merck Serono). Oocyte retrieval was performed 36 hours later.

Oocyte retrieval, intracytoplasmic sperm injection, and blastocyst culture procedures were performed following established standard protocols.

### 2.4 Outcomes

The primary outcome measure was defined as the mean number of oocytes obtained after LPS.

The secondary outcomes variables were the number of follicles >15 mm on triggering day, number of MII oocytes, inseminated MII oocytes, fertilized oocytes, fertilization rate, number of blastocysts, blastulation rate per inseminated MII oocyte, gonadotrophin total dose and duration of the stimulation in days.

Additional exploratory outcomes are also provided comparing follicular phase stimulation and luteal phase stimulation results and the oocyte retrieval rate (oocyte retrieval rate = the number of cumulus-oocyte complex retrieved/the number of follicles *follicles ≥* 15 present on the triggering day). **Supplementary Tables 1**, **2**. The result of the subcategorization of patients according to their diagnosis is also given to check if the onset of stimulation was uniform. Thus, we subcategorized patients whether or not they had a diagnosis of low ovarian reserve according to Bologna criteria. **Supplementary Table 3**.

### 2.5 Statistics

A descriptive analysis of the different variables was carried out. Categorical variables were presented as number of cases and percentage. Numerical variables were presented as number of cases, mean and standard deviation. The normality test was done on these variables using the Shapiro-Wilk test. Kruskal-Wallis rank sum test was used to make the comparison between the groups. R Statistical Software version 4.0.3 (The R Foundation) and Statistical Package for the Social Sciences (SPSS) software (version 20.0, SPSS, Inc, Chicago, IL, USA) were used for statistical analysis.

## 3 Results

### 3.1 Patient characteristics

The study included 541 DuoStim cycles. 186 patients started LPS between 0 and 2 days after oocyte retrieval, 201 patients on the third day, 83 patients on the fourth day and 71 between 5- and 6-days after retrieval.

Overall mean age was 38.57 years. There were no significant differences in AMH level (p=0,7), age (p=0,5) and antral follicle count (p=0,4). (**Table 1**).

**Table 1:** Patient characteristics.

Characteristics^1^	0-2 days (n=186)	3 days (n=201)	4 days (n=83)	5-6 days (n=71)	Overall (n=541)	p value ^2^
**Age**	38.75 ± 3.19	38.66 ± 3.39	38.62 ± 3.22	37.93 ± 3.72	38.59 ± 3.34	0.5
**Anti-Mullerian hormone (pmol/l)**	6.94 ± 5.43	8.03 ± 6.44	8.20 ± 12.55	7.09 ± 5.31	7.54 ± 7.20	0.7
**Antral follicle count**	7.35 ± 3.92	8.60 ± 5.88	8.20 ± 4.82	7.69 ± 4.14	7.98 ± 4.90	0.4

^1^Values are mean +- SD except of p value.

^2^Kruskal-Wallis rank sum text.

### 3.2 Outcomes

#### 3.2.1 Primary outcome measure

The overall mean number of oocytes retrieved was 5.40. The number of oocytes obtained did not differ significantly between the groups (5.12 vs. 5.39 vs. 5.61 vs. 5.89, p=0,6).

#### 3.2.2 Secondary endpoints

Secondary variables such as the number of MII, the number of inseminated MII oocytes, the number of oocytes per gonadotrophin unit, the number of follicles >15 mm on triggering day and the total dose of gonadotrophins were similar in all groups. The study outcomes are shown in **Table 2**. The category of “inseminated MII oocytes” in **Table 2** includes only those patients who decided to proceed with insemination, excluding those who opted to freeze their eggs for later use. This selection process helps explain the higher mean number of inseminated eggs compared to the total number of women after retrieval in the luteal phase.

**Table 2:** Study outcomes.

Variables^1^	0-2 days (n=186)	3 days (n=201)	4 days (n=83)	5-6 days (n=71)	Overall (n=541)	p value ^2^
**Oocytes (n)**	5.12 ± 3.56	5.39 ± 3.74	5.61 ± 3.94	5.89 ± 3.92	5.40 ± 3.73	0.6
**MII Oocytes (n)**	4.28 ± 3.23	4.46 ± 3.29	4.70 ± 3.66	4.65 ± 3.28	4.46 ± 3.33	0.8
**Oocytes per gonadotrophin unit (n)**	1.78 ± 1.39	1.93 ± 1.65	2.09 ± 2.24	1.93 ± 1.35	1.90 ± 1.63	0.7
**Follicles >15 mm on triggering day**	5.40 ± 3.27	6.15 ± 3.88	6.59 ± 5	6.41 ± 4	5.99 ± 3.91	0.2
**Total FSH + menotropin dose (IU)**	3053.23 ± 820.34	3093.22 ± 840.24	3170.51 ± 899.61	3177.82 ± 867.56	3102.46 ± 845.49	0.4
**Duration of stimulation (days)**	10.42 ± 2.31	10.68 ± 2.37	11.27 ± 2.40	10.65 ± 2.37	10.68 ± 2.36	0.033
Inseminated MII oocytes (n)^(^*^)^	4.73 ± 3.32	5.25 ± 3.30	5.52 ± 3.83	5.59 ± 3.47	5.14 ± 3.42	0.3
**Fertilized oocytes (n)**	3.36 ± 2.80	3.95 ± 2.53	4.03 ± 2.73	4.48 ± 3.11	3.80 ± 2.75	0.036
**Fertilization rate per oocyte retrieved (%)**	68.70 ± 30.19	75.73 ± 23.10	73.53 ± 25.29	80.34 ± 21.51	73.37 ± 26.27	0.14
**Blastocysts (n)**	1.82 ± 1.74	2.13 ± 1.61	2.33 ± 2.06	2.91 ± 2.39	2.14 ± 1.85	0.030
**Blastulation rate per MII (%)**	36.35 ± 27.20	44.14 ± 28.42	38.72 ± 24.96	51.06 ± 29.65	41.28 ± 27.93	0.026

^1^Values are mean +- SD except of p value.

^2^Kruskal-Wallis rank sum text.

(*) Includes only patients proceeding with insemination, excluding those who opted to cryopreserve their eggs for additional oocyte banking (n=388). Yellow shading was used to highlight the statistical significant results.

**Figure 2:**
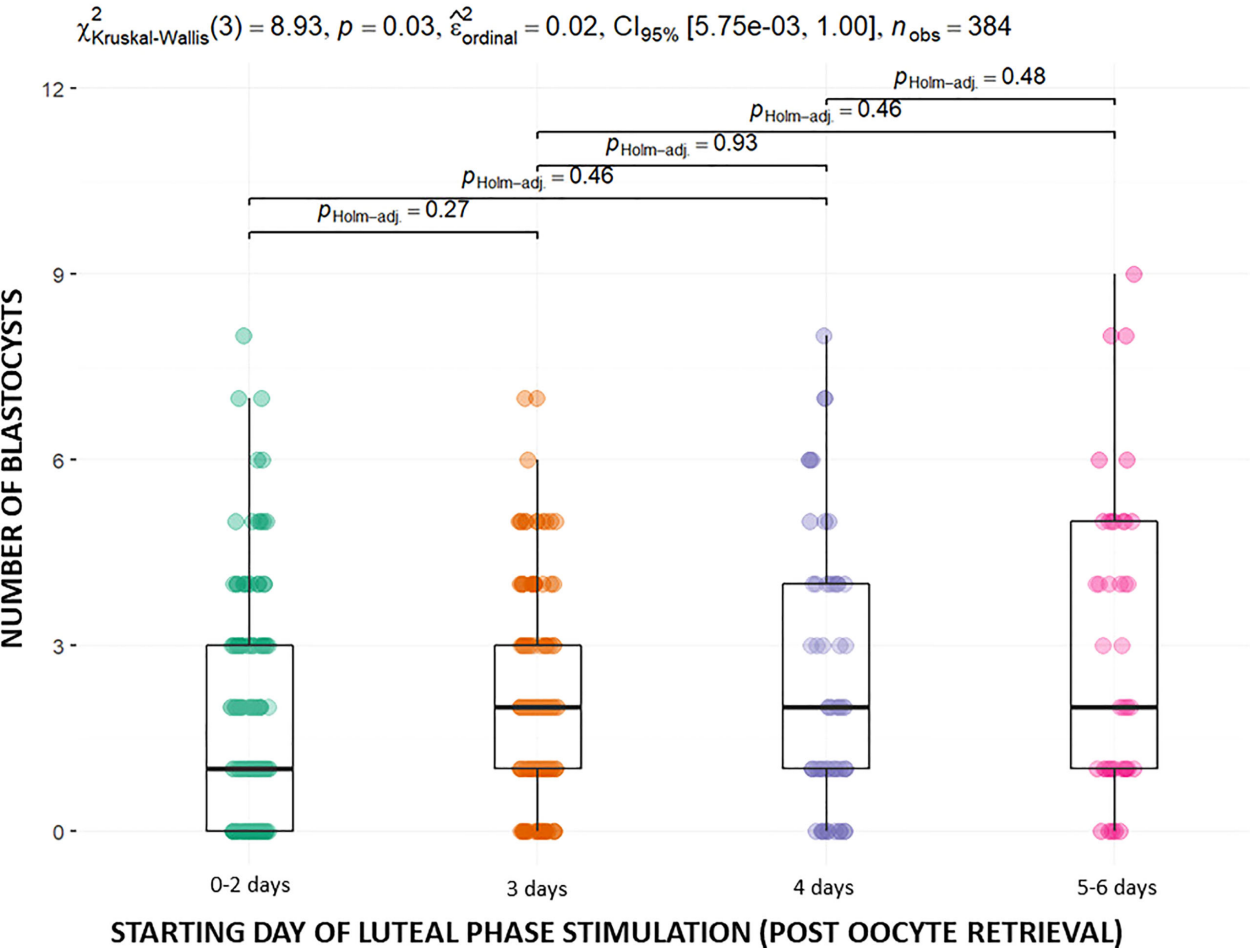
Mean number of blastocysts in the different groups.

In the 4-days subgroup the duration of the stimulation was longer compared to the other subgroups (p<0,05). There was also a significant difference in the number of fertilized oocytes between the groups, which was higher in the 4-days group and 5-6 days group (p<0,05) compared to the other two groups. Evaluating the blastulation rate per fertilized oocyte, we observed a higher rate in the 5-6 days group. This difference was statistically significant. In addition, an increase in the number of blastocysts was observed in this group. Thus, the mean number of blastocysts in the 5-6 days group was 2.91, while in 0-2 days, 3-days and 4-days groups, was 1.82, 2.13 and 2.33 (p<0,05) respectively. (**Figure 2**).

## 4 Discussion

The aim of this study was to investigate whether the timing of LPS onset in DuoStim cycles influences ovarian response. To our knowledge, this is the first study that explores ovarian response by taking into account the days between oocyte retrieval and the start of luteal phase stimulation. The results show no differences in the number of oocytes obtained between the groups, which was our primary outcome. In addition, we find similar results between groups regarding the number of MII and the number of follicles on the trigger day.

When evaluating the secondary variables, we observe unexpected results in terms of oocyte competence between the different groups. In this regard, the number of fertilized oocytes is higher in the groups in which luteal stimulation began after the third day, with a number of MII oocytes that were inseminated similar between groups. In addition, the number of blastocysts and the blastulation rate is higher when stimulation started after the fourth day after retrieval. These differences are not only statistically significant, but also clinically relevant. For instance, there is a difference of 1.09 blastocysts between the 0-2nd day group and the 5th-6th day group.

The ideal time to wait after the oocyte retrieval to start LPS is unknown. Our findings indicate that the response to stimulation does not vary depending on the time of onset. This is consistent with the available evidence, that shows a similar o higher response in the LPS compared to FPS among the different studies despite the variability in the day that LPS begins. In fact, the starting of second stimulation has been reported as early as the day of the retrieval or the following day (3, 5). However, most authors start LPS around the fourth or fifth day post oocyte retrieval (4, 6–8).

Fatemi et al., found that after the administration of a GnRH agonist for the trigger, the luteal phase is very short, with a different endocrine profile between day one and day five post agonist trigger (13). However, the luteal phase physiology has not been fully elucidated and further investigation is necessary. Nevertheless, various theories attempt to explain the reason for the larger cohorts of oocytes in LPS found in previous studies. First, the high level of estradiol and progesterone after FPS, synchronizes the cohort of antral follicles that will grow during LPS, and increases FSH receptors on granulosa cells, resulting in a better response (14).Likewise, a flare-up effect derived from GnRH agonist trigger in the FPS induces a down regulation in the expression of AMH, increasing the number of follicles with a 2-4 mm diameter recruited in the LPS (14, 15). Lastly, FPS may affect the subsequent ovarian microenvironment. In fact, in animal models an increase in angiogenic factors after FPS has been suggested, which enhance the sensitivity of the granulosa cells to FSH within the follicles recruited in the second stimulation (16). Well-designed studies are needed to study whether this improvement in LPS response is of equal intensity regardless of when stimulation begins.

Despite these theories, the biological characteristics of LPS-derived oocytes and their microenvironment are not well understood. Investigating the difference in physiology between follicular waves can provide insights to understand oocyte quality and competence. The study of cumulus cells and follicular fluid is elucidating certain characteristics of oocytes. In fact, different miRNAs patterns have been associated with fertilization success and embryo morphological quality (17, 18). In line with this, a study focused on the comparison of miRNAs profiles between FPS and LPS follicles of women undergone DuoStim, found similar miRNomic signatures from the follicles retrieved after FPS and those retrieved after LPS (19). Nevertheless, other authors suggest that the DuoStim approach leads to changes in the follicular environment, affecting cumulus cell gene expression in luteal-phase-derived oocytes (20). Correlation between these findings and oocyte maturation, fertilization rate or blastulation rate is still unknown.

Our study found a similar ovarian response regardless of the starting day. An apparent better competence of luteal phase oocytes was observed when LPS was initiated above 4 days after retrieval, with a higher number of fertilized eggs and blastocysts. These differences are not explained by key factors in blastulation rate such as age, as there are no differences in this variable between the different groups studied. Nevertheless, the primary limitation of this study is its retrospective nature, which may result in the inadvertent inclusion of residual confounding variables and chance findings. Therefore, the data should be approached with caution. To stablish conclusive findings, additional corroborative prospective studies that randomize the start of the second stimulation are required. This may be useful in developing a consensus regarding DuoStim in terms of timing, dose and type of gonadotrophins in LPS.

## Data availability statement

The raw data supporting the conclusions of this article will be made available by the authors, without undue reservation.

## Author contributions

Authors’ roles: AF, CG-A, RR, JC, JG, AB, RB. Study conception and design, analysis and interpretation of data, writing the article and critical review of the article. AF, CG-A, MH: collection of data. JO: analysis and interpretation of data and critical review of the article. AB, and RB: critical review of the article. All authors contributed to the article and approved the submitted version.
