# Supplementary material for: Influence of the starting day of luteal phase stimulation on double stimulation cycles
Source: Front Endocrinol (Lausanne). 2023 Jul 13;14:1216671. doi: 10.3389/fendo.2023.1216671 (PMC10390300; doi:10.3389/fendo.2023.1216671)
Supplement: Supplementary file 1 [file Table_1.docx]

Supplementary table 1. Comparison of follicular phase stimulation and luteal phase stimulation results.

| Variables^1^ | Follicular phase  (541) | Luteal phase  (541) | Overall  (1082) | p value ^2^ |
| --- | --- | --- | --- | --- |
| Oocytes (n) | 4.18 ± 3.05 | 5.40 ± 3.73 | 4.83 ± 3.45 | <0.001 |
| MII Oocytes (n) | 3.29 ± 2.50 | 4.46 ± 3.33 | 3.91 ± 3 | <0.001 |
| Oocytes per gonadotrophin unit (n) | 1.74 ± 1.64 | 1.90 ± 1.63 | 1.82 ± 1.64 | 0.016 |
| Follicles >15 mm on triggering day | 4.96 ±3.11 | 5.99 ±3.91 | 5.48 ± 3.57 | <0,001 |
| Total FSH + menotropin dose (IU) | 2737.77 ± 818.11 | 3102.46 ± 845.49 | 2923.95 ± 852.15 | <0.001 |
| Duration of stimulation (days) | 9.54 ± 2.02 | 10.68 ± 2.36 | 10.11 ± 2.28 | <0.001 |
| Fertilized oocytes (n) | 2.20 ± 1.98 | 3.80 ± 2.75 | 3.01 ± 2.53 | <0.001 |
| Fertilization rate per oocyte retrieved (%) | 73.01 ± 32.23 | 73.37 ± 26.27 | 73.21 ± 29.21 | 0.2 |
| Blastocysts (n) | 0.85 ± 1.12 | 2.14 ± 1.85 | 1.50 ± 1.67 | <0.001 |

^1^Values are mean +- SD except of p value. ^2^Kruskal-Wallis rank sum text
