# Supplementary material for: Influence of the starting day of luteal phase stimulation on double stimulation cycles
Source: Front Endocrinol (Lausanne). 2023 Jul 13;14:1216671. doi: 10.3389/fendo.2023.1216671 (PMC10390300; doi:10.3389/fendo.2023.1216671)
Supplement: Supplementary file 2 [file Table_2.docx]

Supplementary Table 2. Oocyte retrieval rate.

| Characteristics^1^ | 0-2 days  (n=186) | 3 days  (n=201) | 4 days  (n=83) | 5-6 days  (n=71) | Overall  (n=541) | p value ^2^ |
| --- | --- | --- | --- | --- | --- | --- |
| Follicular phase oocyte retrieval rate (%) | 92.82 ± 54.26 | 86.67 ± 51.09 | 95.61 ± 49.03 | 76.12 ± 36.84 | 88.73 ± 50.49 | 0.040 |
| Luteal phase oocyte retrieval rate (%) | 100.86 ± 60.59 | 92.55 ± 49.27 | 90.90 ± 34.83 | 98.43 ± 51.15 | 95.95 ± 51.95 | 0.7 |

^1^Mean (SD)

^2^Kruskal-Wallis Rank sum test

^1^Values are mean +- SD except of p value. ^2^Kruskal-Wallis rank sum text
