# Supplementary material for: Influence of the starting day of luteal phase stimulation on double stimulation cycles
Source: Front Endocrinol (Lausanne). 2023 Jul 13;14:1216671. doi: 10.3389/fendo.2023.1216671 (PMC10390300; doi:10.3389/fendo.2023.1216671)
Supplement: Supplementary file 3 [file Table_3.docx]

Supplementary Table 3. Subcategorization of patients by diagnosis

| Characteristics^1^ | 0-2 days  (n=185) | 3 days  (n=200) | 4 days  (n=83) | 5-6 days  (n=71) | Overall  (n=539) | p value ^2^ |
| --- | --- | --- | --- | --- | --- | --- |
| Bologna criteria patients (n=190) | 70/185 (37.8%) | 70/200 (35%) | 26/83 (31.32%) | 24/71 (33.80%) | 190/539 (35.25%) | 0.759 |
| No Bologna criteria patients (n=349) | 115/185 (62.2%) | 130/200 (65.0%) | 57/83 (68.7%) | 47/71 (66.2%) | 349/539 (64.74%) | 0.759 |

^1^n/N (%)

^2^Pearson´s Chi-squared test
